# Raffinocyclicin is a novel plasmid-encoded circular bacteriocin produced by *Lactococcus raffinolactis* with broad-spectrum activity against many gram-positive food pathogens

**DOI:** 10.1128/aem.00809-24

**Published:** 2024-08-27

**Authors:** Felipe Miceli de Farias, Paula M. O'Connor, Colin Buttimer, Eleni Kamilari, Maria Cecilia Soria, Crystal Nicole Johnson, Aiswariya Deliephan, Daragh Hill, Oxana Fursenko, Jonathan Wiese, Lorraine A. Draper, Catherine Stanton, Colin Hill, R. Paul Ross

**Affiliations:** 1 APC Microbiome Ireland, University College Cork, Cork, Ireland; 2 Teagasc Food Research Centre, Cork, Ireland; 3 Department of Biochemistry and Microbiology, Oklahoma State University – Center for Health Sciences, Tulsa, Oklahoma, USA; 4 Kraft Heinz Corporate Headquarters, Chicago, Illinois, USA; Universita degli Studi di Napoli Federico II, Portici, Italy

**Keywords:** circular bacteriocin, antimicrobial peptide, raffinocyclicin, antimicrobial activity, *Lactococcus raffinolactis*

## Abstract

This study describes the discovery and characterization of raffinocyclicin, a novel plasmid-encoded circular bacteriocin, produced by the raw milk isolate *Lactococcus raffinolactis* APC 3967. This bacteriocin has a molecular mass of 6,092 Da and contains 61 amino acids with a three-amino acid leader peptide. It shows the highest identity to the circular bacteriocins bacicyclicin XIN-1 (42.62%), aureocyclicin 4185 (42.62%), and garvicin ML (41.53%). A broad inhibitory spectrum includes strains from *Staphylococcus*, *Enterococcus*, *Streptococcus*, *Micrococcus*, *Lactobacillus*, *Leuconostoc,* and *Listeria*, in addition to a pronounced inhibitory effect against *Lactococcus* and *Clostridium*. It displays low sensitivity to trypsin, most likely as a result of its circular nature. The raffinocyclicin gene cluster is composed of 10 genes: 6 core genes, genes encoding an accessory three-component ABC transporter (*rafCDE*), and a putative transcriptional regulator related to the MutR family. A lack of inhibitory activity in the cell-free supernatant combined with the pronounced activity of cell extracts suggests that the majority of raffinocyclicin is associated with the cell rather than being released to the extracellular environment. This is the first report of a bacteriocin produced by the *L. raffinolactis* species.

**IMPORTANCE:**

The present study aimed to characterize raffinocyclicin, a novel circular bacteriocin produced by the lactic acid bacteria *Lactococcus raffinolactis* APC 3967. Bacteriocins are generally cationic and hydrophobic peptides with antimicrobial activity, which present diverse biotechnological properties of interest for the food industry. Raffinocyclicin inhibits a wide range of bacteria, including foodborne pathogens, and is stable against different treatments which suggest its potential as a natural biopreservative. Whole-genome sequencing and the genetic analysis of the raffinocyclicin gene cluster showed that it is encoded by plasmid that could be used in the future to transfer the ability to produce the bacteriocin to other lactic acid bacteria for industrial applications. These results together highlight the potential of this novel antimicrobial as a biopreservative to be used by the food industry.

## INTRODUCTION

One of the main challenges the food industry faces in product development is food preservation. While preservatives such as sorbate, benzoate, and nitrate have been used as chemical preservatives, consumers have expressed a preference for healthier and more natural foods (1). Thermal treatment is an alternative, but a loss of nutritional value, alteration of the organoleptic properties, and generation of carcinogenic products in some food sources restrict the application of this technique (2). One group of microorganisms used for millennia in the preservation of foods through fermentation is the lactic acid bacteria (LAB). This bacterial group comprises several genera, including *Lactococcus*, lactobacilli, *Leuconostoc*, *Pediococcus*, *Streptococcus*, and *Enterococcus*. These bacteria can be found in various fermented foods, including yoghurt, sauerkraut, kefir, and cheese (3). LAB ferment sugars in the food matrix and produce lactic acid that can inhibit spoilage microorganisms, thus extending the shelf life of foods (1). However, this is not the only substance produced by these bacteria with a biopreservative property; many LAB also produce antimicrobial peptides known as bacteriocins (2).

Bacteriocins are defined as ribosomally synthesized multifunctional peptides produced by prokaryotes, which can have either a broad spectrum of antimicrobial activity (targeting a range of species) or a narrow spectrum (targeting closely related species and strains) (4, 5). These peptides are generally cationic and hydrophobic, with the producing strain possessing immunity to its own bacteriocin. A number of classification systems have been proposed (5–8). However, they can be broadly divided into two main classes: class I peptides that undergo post-translational modification, such as circular bacteriocins, and class II unmodified peptides (9). Several studies have reported their potential as biopreservatives (10, 11). Moreover, the class I bacteriocin nisin (E234) has been approved as a biopreservative since 1969.

Bacteriocins produced by LAB offer several biotechnological advantages over chemical preservatives. These include (i) their self-affirmed GRAS (generally recognized as safe) status; (ii) they are generally non-toxic to eukaryotic cells; (iii) resistance to a range of pH, temperature, and food-associated enzymes; (iv) the ability to target pathogens and food spoilage bacteria; and (v) they can be encoded on mobile elements, such as conjugative plasmids or transposons, thus facilitating their mobilization to starter cultures (10, 11). As for their applications in food, four main ways have been described: (i) direct application of the bacteriocin as a pure or partially purified substance; (ii) production in the food matrix by the bacteriocinogenic strain; (iii) incorporation of the antimicrobial in food packaging; and (iv) use of a fermented food ingredient derived from the bacteriocinogenic strain (12–14).

Circular bacteriocins are a group of bacteriocins that have been explored in recent years for their biotechnological potential. To date, this group of bacteriocins is composed of 23 peptides, with altitudin A being the most recently described in 2024 (15–24). They can be divided into two groups: (i) peptides with a high isoelectric point and a high net charge (e.g., enterocin AS-48 and garvicin ML) or (ii) peptides with a considerably lower isoelectric point and a low net charge (e.g., butyrivibriocin AR10 and paracyclicin) (18). One main characteristic that makes circular bacteriocins so promising for food preservation and safety is that they are highly resistant to pH, temperature, and proteolytic action. This characteristic is due to their highly stable circular and globular conformation (25). However, the application of this group of bacteriocins is limited due to the lack of knowledge about their biosynthetic pathways and biopreservative and safety potential compared to other classes.

This study describes the isolation and characterization of raffinocyclicin, a circular bacteriocin produced by the LAB strain *Lactococcus raffinolactis* APC 3967.

## RESULTS

### Antimicrobial spectrum of *Lactococcus raffinolactis* APC 3967

The antimicrobial activity of *L. raffinolactis* APC 3967, a strain isolated from bulk tank milk, was evaluated by the spot-on-lawn assay and showed a broad spectrum of activity, with 22 of 31 indicator strains inhibited by the producer (Table 1). Sensitive strains included members of the following genera: *Staphylococcus*, *Enterococcus*, *Streptococcus*, *Micrococcus*, *Lactococcus*, *Lactobacillus*, *Leuconostoc*, *Listeria,* and *Clostridium*. No inhibition was observed against the gram-negative *Escherichia* sp. UCC. The two most sensitive species identified in this screening were *Clostridium perfringens* and *Lactococcus lactis*.

**TABLE 1:** Growth conditions of the indicator strains and inhibitory spectrum of raffinocyclicin in the spot-on-lawn assay^*a*^^,^^*b*^

Species	Strains	Growth condition			Activity
		Temperature (°C)	Atmosphere	Media	
*Staphylococcus epidermidis*	DSM 3095	37	Aerobic	BHI	**+**
S*taphylococcus capitis*	APC 2923	37	Aerobic	BHI	**–**
*Staphylococcus pseudointermedius*	DK 279	37	Aerobic	BHI	**–**
***Staphylococcus aureus***	**DPC 5645**	**37**	**Aerobic**	**BHI**	**–**
***S. aureus***	**C5M**	**37**	**Aerobic**	**BHI**	**–**
***S. aureus***	**B2M**	**37**	**Aerobic**	**BHI**	**+**
***S. aureus***	**A8M**	**37**	**Aerobic**	**BHI**	**-**
*Enterococcus faecalis*	ATCC 29200	37	Aerobic	BHI	**++**
*E. faecalis*	V583	37	Aerobic	BHI	**+**
*Enterococcus faecium*	NCD 0942	37	Aerobic	BHI	**++**
*E. faecium*	DPC 5152	37	Aerobic	BHI	**+**
*E. faecium*	DPC 3675	37	Aerobic	BHI	**++**
*E. faecium*	DSM 26544	37	Aerobic	BHI	**+**
VRE	APC 1026	37	Aerobic	BHI	**++**
VRE	APC 1027	37	Aerobic	BHI	**++**
VRE	APC 1028	37	Aerobic	BHI	**++**
VRE	APC 1029	37	Aerobic	BHI	**++**
*Micrococcus luteus*	APC 4061	37	Aerobic	BHI	**++**
*Streptococcus agalactiae*	35	37	Aerobic	TSA	**++**
*S. agalactiae*	119	37	Aerobic	TSA	**++**
***Lactococcus lactis***	**HP**	**30**	**Aerobic**	**GM17**	**+++**
***L. lactis***	**ATCC 11454**	**30**	**Aerobic**	**GM17**	**+++**
***Lactobacillus delbrueckii* subsp. *bulgaricus***	**LMG 6901**	**37**	**Anaerobic**	**MRS**	**+**
***Leuconostoc mesenteroides***	**KH 24**	**30**	**Aerobic**	**MRS**	**+**
*Listeria innocua*	UCC	37	Aerobic	BHI	**++**
***Listeria monocytogenes***	**EDGe**	**37**	**Aerobic**	**BHI**	**+**
***Clostridium perfringens***	**EM124**	**37**	**Anaerobic**	**RCM**	**+++**
***Bacilllus cereus***	**KH 1453**	**37**	**Aerobic**	**BHI**	**–**
***Escherichia* sp**.	**UCC**	**37**	**Aerobic**	**BHI**	**–**
***Klebsiella pneumoniae***	**C02.b**	**37**	**Aerobic**	**BHI**	**–**
***Pseudomonas aeruginosa***	**PA-01**	**37**	**Aerobic**	**BHI**	**–**

^*a*^ VRE, vancomycin-resistant enterococci; MRS, de Man, Rogosa, and Sharpe; BHI, brain-heart infusion; RCM, reinforced clostridial media; GM17, glucose M17; –, no activity; +, 0.5–5 mm inhibition zone; ++, >5–≤10 mm inhibition zone; +++, >10 mm inhibition zone.

^*b*^ Related species or genera to foodborne pathogens and food spoilage bacteria were highlighted in bold.

### Sensitivity of raffinocyclicin to proteases and NaOH

The antimicrobial activity of the strain *L. raffinolactis* APC 3967 was resistant to NaOH 0.2 M, partially sensitive to trypsin (retaining 73% of the initial activity after treatment), and highly sensitive to proteinase K (retaining only 7% of the initial activity after treatment), confirming a proteinaceous nature (Table 2).

**TABLE 2:** Residual antimicrobial activity after the different treatments^*a*^

Treatment	Phenotype	Residual activity
Control	N.A.	100%
NaOH 0.2 M	R	100%
Proteinase K	S	7%
Trypsin	PS	73%

^*a*^ R, resistant; PS, partial sensitive; S, sensitive; N.A., not applicable.

### Colony mass spectrometry

**Fig 1:**
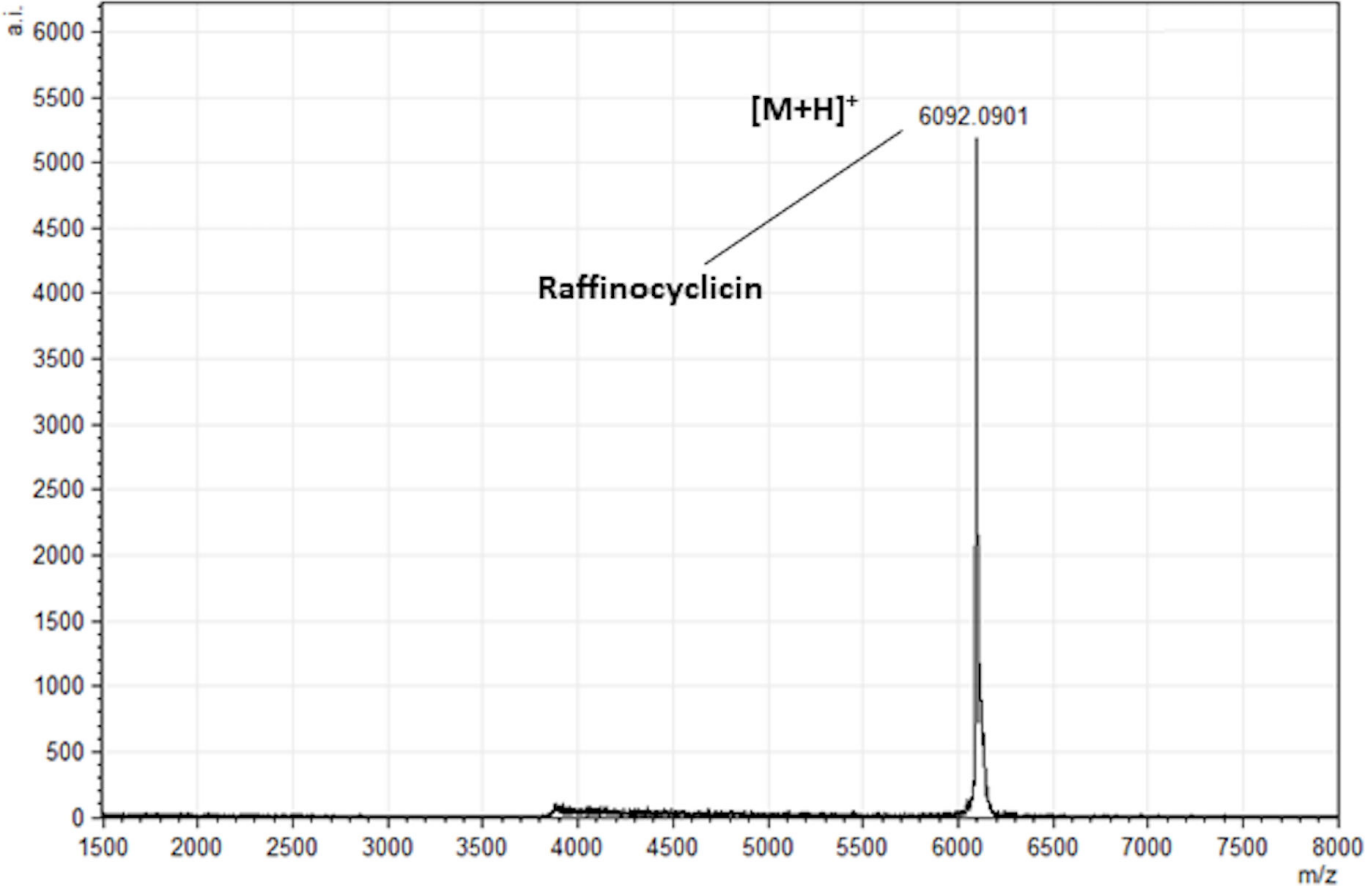
Colony matrix-assisted laser desorption/ionization coupled to time-of-flight mass spectrum revealing the molecular mass of raffinocyclicin produced by *L. raffinolactis* APC 3967 (6,092 Da).

The observed molecular mass of 6,092 Da did not match the mass of any known bacteriocin, suggesting that the antimicrobial activity was due to a potentially novel peptide (Fig. 1).

### Genomic analysis

**Fig 2:**
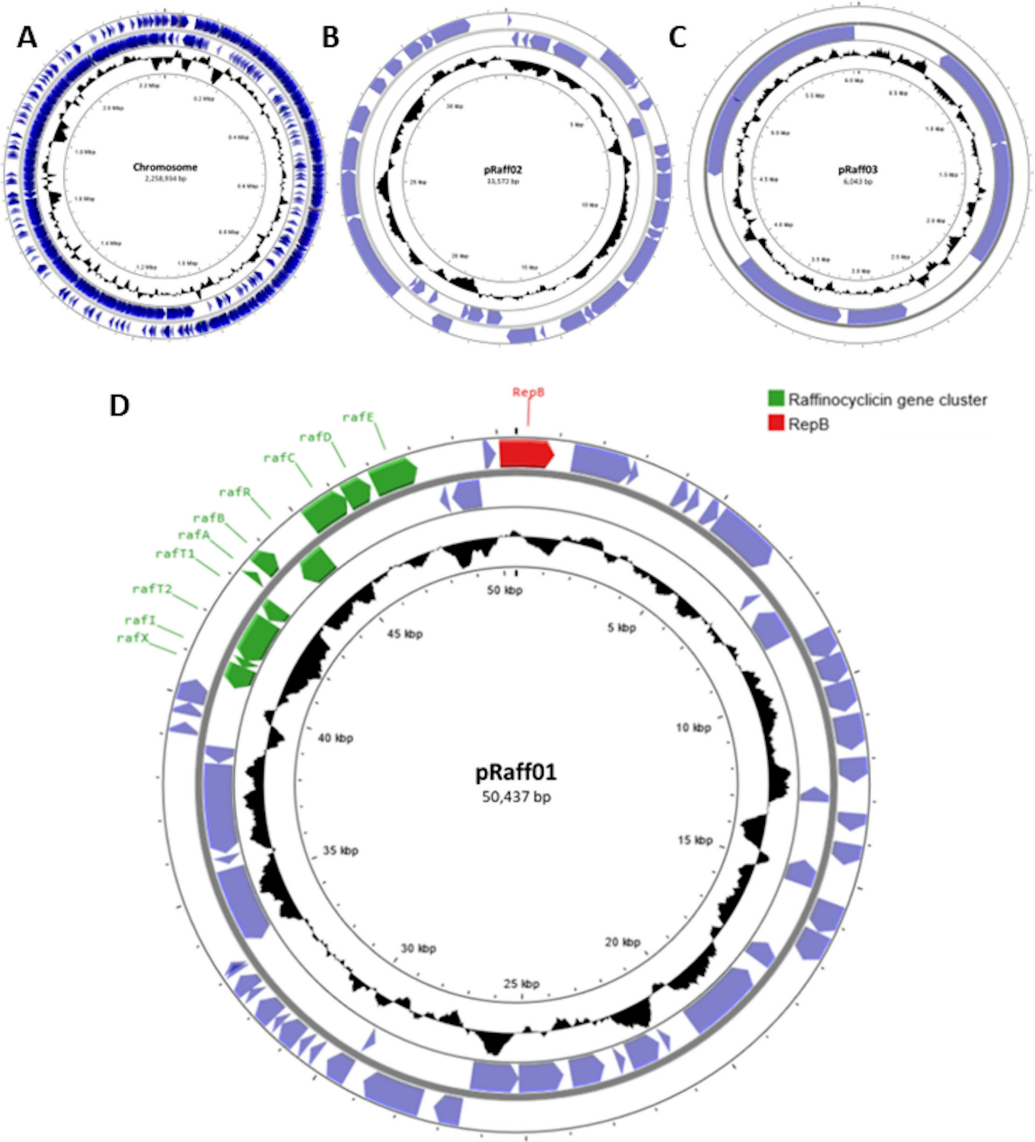
Genomic map of *L. raffinolactis* APC 3967. Chromosome (**A**); plasmid pRaff02 (**B**); plasmid pRaff03 (**C**); and plasmid pRaff01 (**D**). In green are all genes presented in the gene cluster of raffinocyclicin. The protein RepB is highlighted in red. The maps were created using Proksee (https://proksee.ca/).

To identify the gene(s) involved in the production of the antimicrobial molecule, the whole genome of *L. raffinolactis* APC 3967 was sequenced. The assembly of long and short reads resulted in a complete genome with a size of 2,348,986 bp (sequence coverage: short reads 50×, long reads 35×) with a GC content of 39.7%. The genome consists of a chromosome (2,258,934 bp) and three plasmids (50,437, 33,572, and 6,043 bp; Fig. 2). These extrachromosomal DNA elements harbor genes associated with plasmid functions, including a RepB family replication protein and/or other proteins, confirming their plasmid-like nature.

After annotation using Prokaryotic Genome Annotation Pipeline (PGAP), the genome was analyzed using BAGEL4 and Antismash 7.0 for the presence of bacteriocin gene clusters. Two gene clusters were found: (i) one related to a putative class II lactococcin 972-like bacteriocin, found with both programs and (ii) a circular garvicin ML-related bacteriocin, found only with BAGEL4. The lactococcin 972-like bacteriocin is encoded on the chromosome, and the putative circular bacteriocin is encoded on the ~50-kb plasmid pRaff01 (Fig. 2).

**Fig 3:**
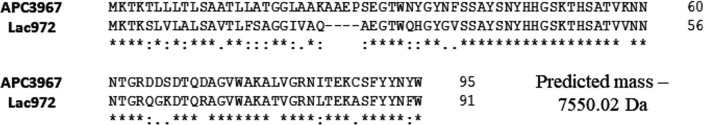
Alignment of the precursor peptides of the putative bacteriocin and lactococcin 972, the leader sequences are underlined. Gap (−), identical amino acids (*), conservative substitutions (:) and weak conservative substitutions (.).

**Fig 4:**
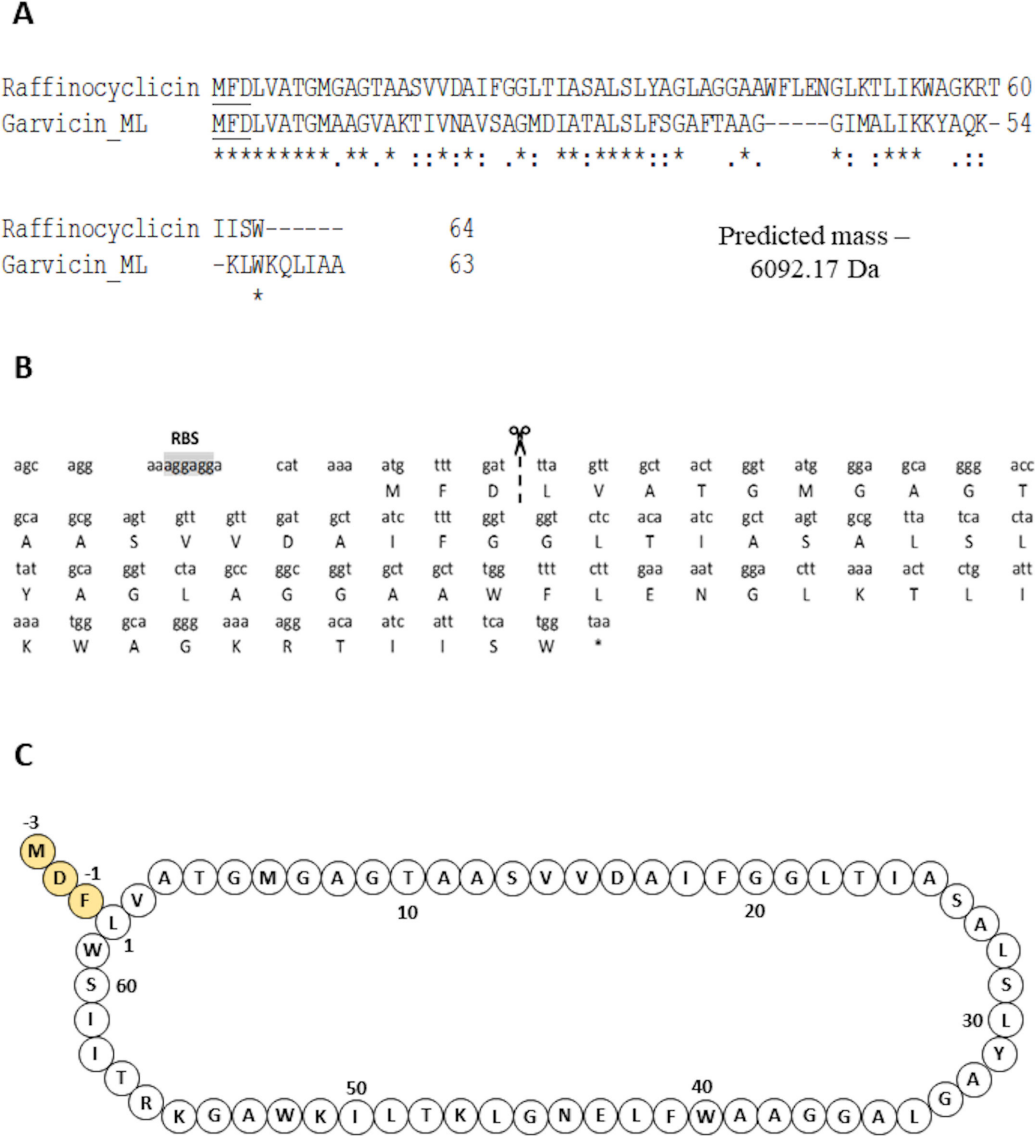
(**A**) Alignment of the precursor peptides of raffinocyclicin and garvicin ML, the leader sequences are underlined. Gap (−), identical amino acids (*), conservative substitutions (:), and weak conservative substitutions (.). The leader sequences are underlined. (**B**) The nucleotide sequence of the raffinocyclicin propeptide. The cleavage site is highlighted by the dashed line between residues 3 and 4. RBS, putative ribosome-binding site—7 bp upstream of the start codon. *, stop codon. (**C**) Predicted circular structure of raffinocyclicin. The yellow amino acids correspond to the leader sequence of the bacteriocin.

To determine if the strain produces either or both putative bacteriocins, the theoretical masses of their core peptides were compared with the 6,092 Da mass obtained from colony mass spectrometry. Based on the cleavage site of the closely related lactococcin 972, the lactococcin 972-like bacteriocin was predicted to be 7550.02 Da (26) (Fig. 3). Using the cleavage site of garvicin ML, the core peptide of the putative circular bacteriocin was calculated as 6110.17 Da (27) (Fig. 4A). However, circular bacteriocins undergo a loss of 18 Da after the head-to-tail cyclization, between leucine 1 and tryptophan 61 in this instance, culminating in a predicted final mass of 6092.17 Da. This precisely matches the mass detected by the colony mass spectrometry, confirming that the strain is producing a novel circular bacteriocin herein named raffinocyclicin. Therefore, subsequent analysis focused on this bacteriocin. The nucleotide sequence of the surrounding sequence reveals the presence of the canonical ribosome-binding site AGGAGG placed appropriately upstream of the structural gene for raffinocyclicin in the operon (Fig. 4B). A graphical representation of the core peptide is presented in Fig. 4C.

**Fig 5:**
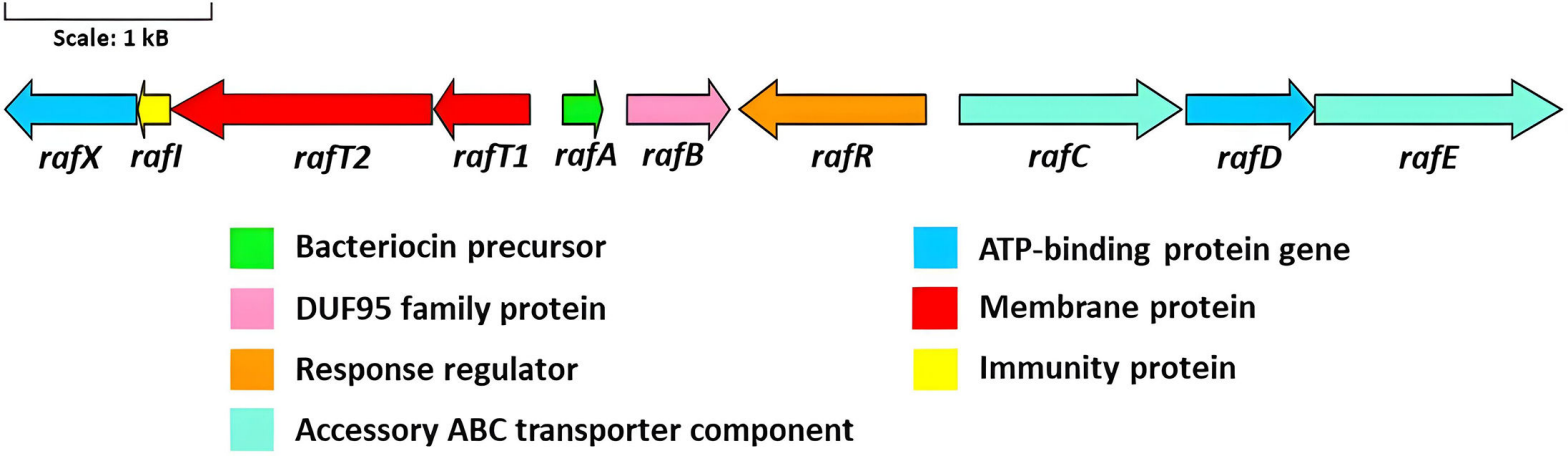
The proposed gene cluster of raffinocyclicin.

*In silico* analysis of pRaff01 revealed that the novel circular bacteriocin gene cluster is ~7.5 kb in length and is composed of 10 genes (*rafX*, *rafI*, *rafT2*, *rafT1*, *rafA*, *rafB*, *rafR*, *rafC*, *rafD*, *rafE*) across both DNA strands (Fig. 2D and 5). The principal physiochemical characteristics, subcellular localization, and highest similarity hits of each gene product are described in Table 3. When compared to other circular bacteriocins, the raffinocyclicin gene cluster contains more genes than previously described clusters for similar bacteriocins. This is due to the presence of a second putative membrane protein and an accessory multi-component ABC transporter. The same genes can be found in the gene clusters of aureocyclicin 4185, carnocyclin A, enterocin AS-48, and garvicin ML. With the exception of garvicin ML, which has 9 genes, the other 3 circular bacteriocins have gene clusters comprising 10 genes.

**TABLE 3:** Bioinformatics analysis of the raffinocyclicin gene cluster

Gene	No. of aa	Similarity (reference)	Localization (confidence) by PSORTb	ProtParam results				Putative function
				p*I*	*M*_*w*_	GRAVY	AI	
*rafX*	212	ATP-binding cassette domain-containing protein [*Lactococcus raffinolactis*] (WP_096824116.1)	Cytoplasmatic (7.5)	5.37	24,603	−0.106	101.56	ABC transporter ATPase
*rafI*	52	No similarity with other protein	Membrane (9.55)	9.82	5,774	1.590	187.50	Immunity protein
*rafT2*	421	Hypothetical protein RR45_GL001457 [*Lactococcus chungangensis* CAU 28 = DSM 22330] (PCR99707.1)	Membrane (10.0)	9.78	50,459	0.906	141.62	Membrane protein
*rafT1*	154	Hypothetical protein [*Lactococcus raffinolactis*] (WP_096824076.1)	Membrane (10.0)	9.96	17,588	0.996	146.82	Membrane protein
*rafA*	64	Multispecies: hypothetical protein [*Lactococcus*] (WP_031366965.1)	Membrane (9.55)	9.53	6,092	0.872	117.05	Raffinocyclicin precursor
*rafB*	165	Stage II sporulation protein M [*Lactococcus raffinolactis*] (QIW57245.1)	Membrane (10.0)	8.97	18,937	1.067	150	Membrane protein DUF95
*rafR*	301	Positive transcriptional regulator, MutR family [*Lactococcus raffinolactis*] (PCS09761.1)	Cytoplasmatic (7.5)	5.12	34,830	−0.292	92.89	Response regulator protein
*rafC*	358	Efflux RND transporter periplasmic adaptor subunit [*Lactococcus raffinolactis*] (WP_172507128.1)	Unknown (3.33)	8.60	39,688	−0.400	94.36	ABC transporter component
*rafD*	208	ABC transporter ATP-binding protein [*Lactococcus raffinolactis*] (WP_096824080.1)	Membrane (9.96)	5.60	22,868	0.092	105.34	ABC transporter ATPase
*rafE*	398	ABC transporter permease [*Lactococcus raffinolactis*] (WP_172507129.1)	Membrane (10.0)	9.28	44,398	0.029	107.44	ABC transporter component

The gene *rafA* encodes the core peptide of the bacteriocin of this genetic cluster, which is 64 amino acids in length and includes a 3-amino acid leader sequence and a mature peptide of 61 amino acids. This leader sequence (MFD) is the same as that of garvicin ML, which is a closely related circular bacteriocin. The gene *rafB* encodes a membrane protein that contains the DUF95 domain of unknown function. This type of protein has been found in all circular bacteriocin gene clusters described in the literature (15–24). The gene *rafR*, on the opposite DNA strand, encodes a positive transcriptional regulator belonging to the MutR family, which could potentially be involved in regulating raffinocyclicin biosynthesis. The genes *rafC*, *rafD,* and *rafE* are thought to encode a three-component ABC transporter often found in the genetic clusters of the circular bacteriocins (aureocyclicin 4185, carnocyclin, circularin A, enterocin AS-48, and garvicin ML) with a putative role in transport and immunity (18). Four genes were found upstream of the core peptide: *rafT1*, *rafT2*, *rafI*, and *rafX*. The two genes *rafT1* and *rafT2* encode two putative membrane proteins with low similarity to other membrane proteins associated with circular bacteriocins. The gene *rafI* encodes a putative dedicated immunity protein with a short, hydrophobic, and cationic product of 52 amino acids. These biochemical characteristics are shared between the immunity protein of all circular bacteriocins (18). Lastly, the gene *rafX* encode*s* a cytoplasmatic ATP-binding cassette domain-containing protein.

### Mature peptide alignment

**Fig 6:**
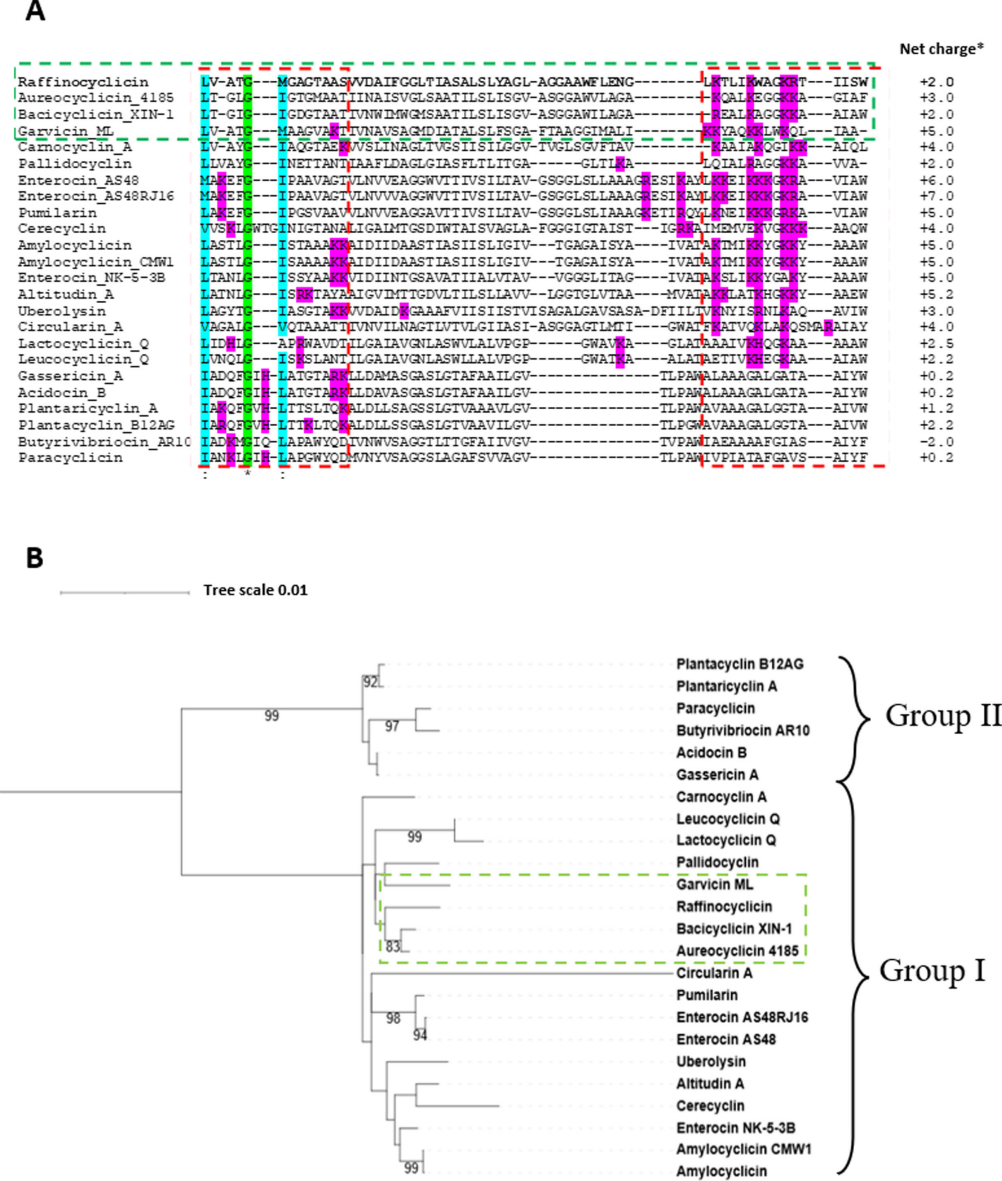
(**A**) Amino acid alignment of the mature circular bacteriocins (MAFFT v7.4.89). Gap (−), identical amino acids (*), and conservative substitutions (:). Dashed red lines are used to show the region of the peptide where most of the positively charged amino acids are found (H, K, and R). (**B**) Maximum likelihood phylogenetic tree of the mature circular bacteriocins using MAFFT for the alignment. Bootstrapped *1,000 replicates. The green boxes were used to highlight raffinocyclicin, garvicin ML, bacicyclicin XIN-1, and aureocyclicin 4185. *, net charge predicted for the bacteriocin linear form using GeneScript Peptide Property Tool.

The mature peptide amino acid sequences of all 23 circular bacteriocins identified to date were aligned with the mature sequence of raffinocyclicin (Fig. 6A). To improve the alignment quality, the first three amino acids of the group II bacteriocins were permuted to the end of the sequence as previously described (20). The change of the amino acid position does not change the relevance of the alignment due to the circular nature of these peptides. The homology between peptides is very low. Assuming that the numbering of amino acids remains the same after circularization, except for group II, the first amino acid of all circular peptides is hydrophobic in nature (valine, methionine, leucine, or isoleucine). Another observed characteristic is that the last amino acid of most peptides is aromatic (phenylalanine, tyrosine, and tryptophan), with the only exceptions being carnocyclin A, garvicin ML, and pallidocyclin that have leucine, alanine, and alanine, respectively. Lastly, most of the positively charged amino acids (lysine, arginine, and histidine) of all circular bacteriocins seem to cluster together in just one region of the peptide.

The phylogenetic tree of the mature core peptides reveals that the closest relatives of raffinocyclicin are bacicyclicin XIN-1 and aureocyclicin 4185 (both with 42.62% identity), followed by garvicin ML (41.53% identity; Fig. 6B). It is essential to highlight that group I and group II form two distinct branches in the phylogenetic tree.

### Bacteriocin purification

**Fig 7:**
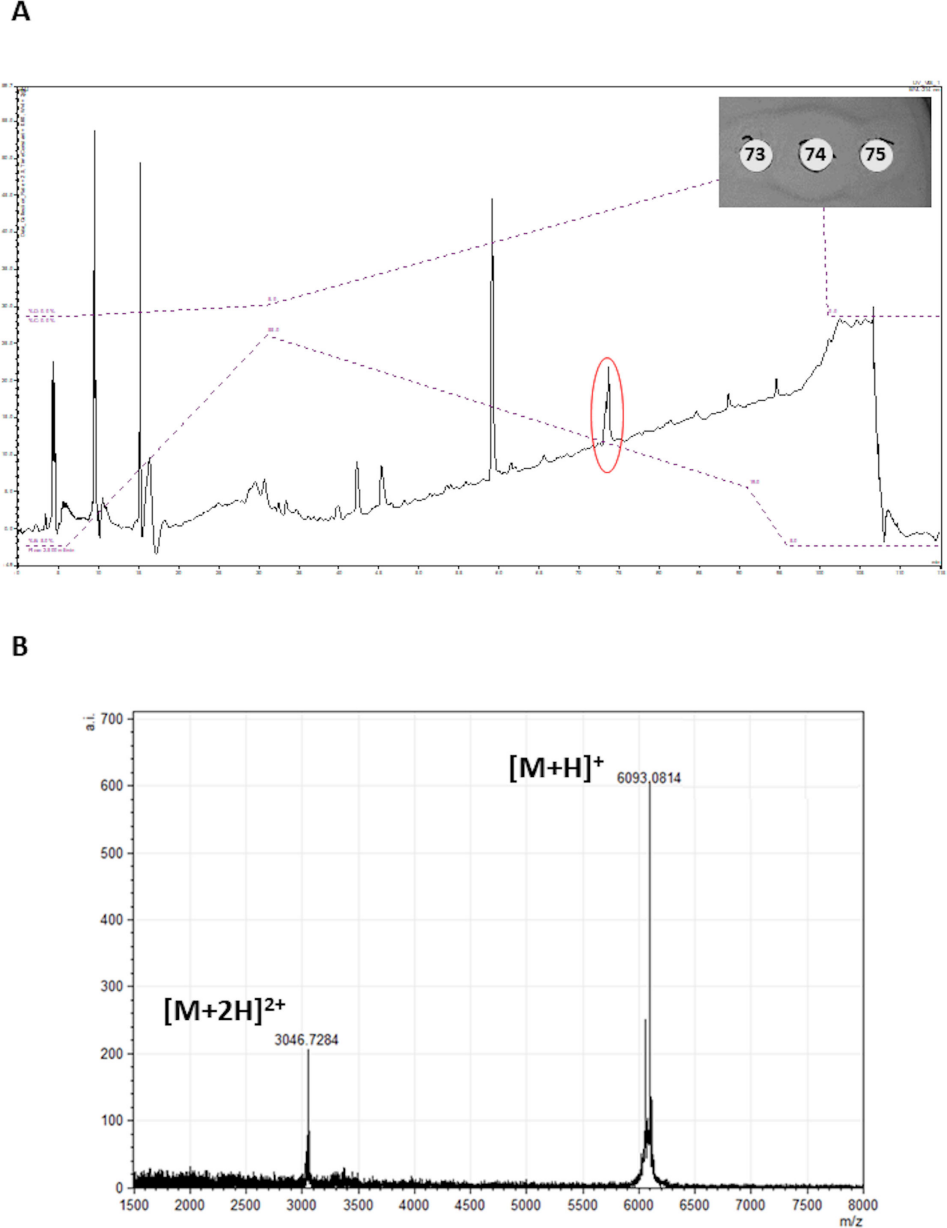
(**A**) HPLC analysis of the cell extract of *L. raffinolactis* APC 3967 with fraction 74, highlighted by the red circle, and showing antimicrobial activity against *L. lactis* HP. (**B**) Detection of the theoretical mass of raffinocyclicin by MALDI-TOF mass spectrum from fraction 74. Expected mass: 6,092.17 Da (±1 Da).

In order to confirm that the molecular mass found by colony mass spectrometry was responsible for the antimicrobial activity observed in the spot-on-lawn assay, the peptide was purified by a two-step protocol. It is important to highlight that the cell extract was used since no inhibitory activity was observed in the cell-free supernatant (CFSN). Following extraction from the cell pellet using IPA, the peptide was semi-purified by solid-phase extraction (SPE) and then fractionated on a semi-preparative Jupiter C4 RP-HPLC column. The presence of the antimicrobial activity was monitored by testing the fractions obtained against *L. lactis* HP by well diffusion assay. The results reveal that fraction 74 obtained from HPLC presents the highest activity against the indicator strain (Fig. 7A). This finding correlates with the peak (~ 15 mV)—outlined in red on the chromatogram. MALDI-TOF (matrix-assisted laser desorption/ionization coupled to time-of-flight) analysis of the active fraction reveals the presence of a peptide with the expected molecular mass for raffinocyclicin. Both doubly charged [M + 2H]^2+^ and singly charged [M + H]^+^ species of the peptide are present with masses of 3046.72 Da (±1 Da) and 6093.08 Da (±1 Da), respectively (Fig. 7B).

### Pangenomic analysis

Pangenome analysis for the strain was performed to investigate the phylogenetic relationship of the genome of *L. raffinolactis* APC 3967 with other publicly available *L. raffinolactis* genomes described in the literature. The strain *L. laudensis* DSM 28961, a representative of a closely related species, was used as an outlier for this *in silico* analysis. First, all genomes were annotated with Prokka, with the resulting output subjected to pangenome analyses using Roary using a minimum percentage of amino acid identity for a positive hit at 95%. Those genes present in all genomes were considered core genes.

**Fig 8:**
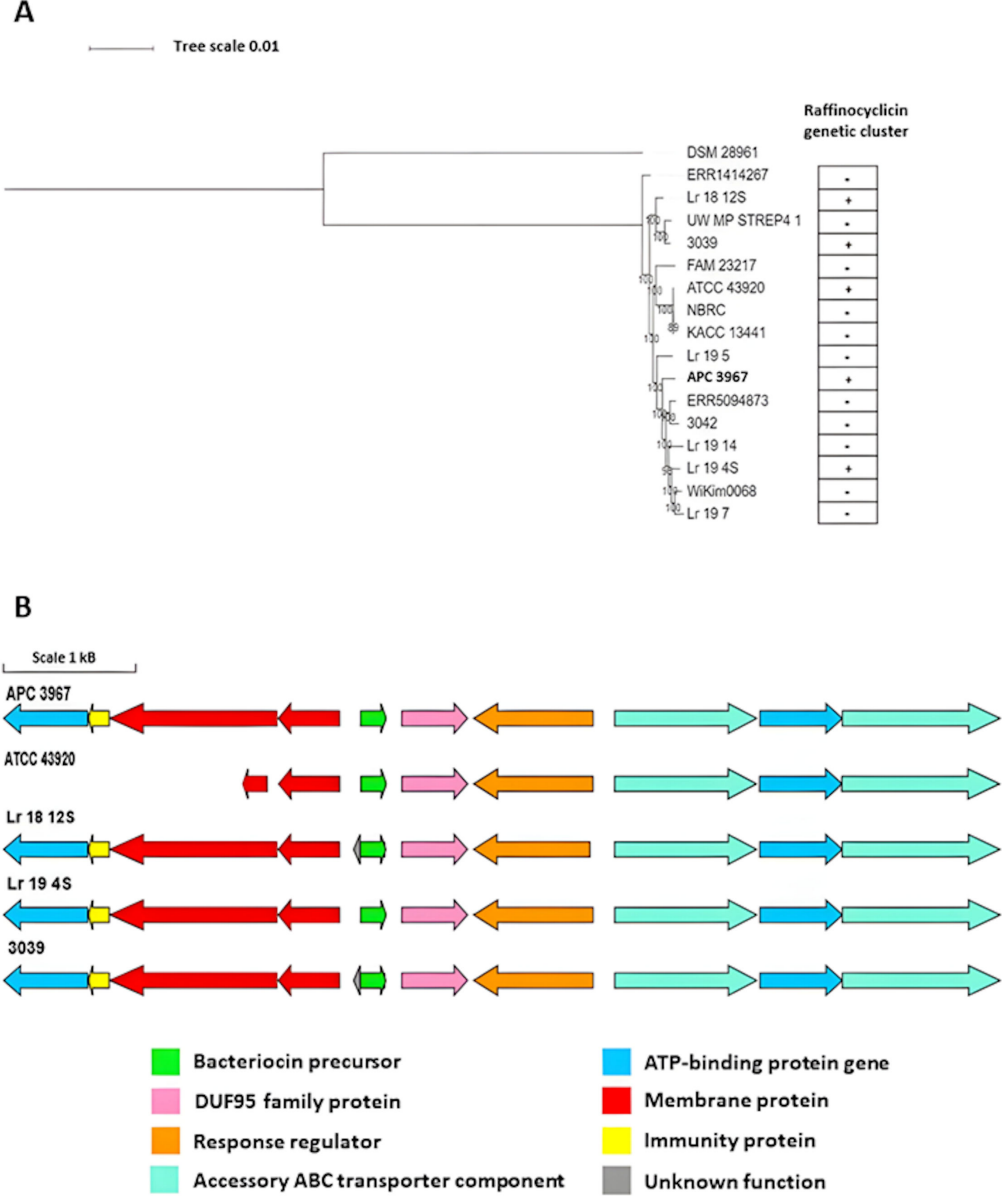
(**A**) Approximate maximum likelihood phylogenetic tree of non-redundant genomes belonging to *L. raffinolactis*. The tree showing all 17 genomes (16 *L*. *raffinolactis* and 1 *L. laudensis*) was computed using a core gene alignment file generated using Roary, bootstrapped *1,000 replicates. The strain *L. laudensis* DSM 28961 was used as an outlier. (**B**) Raffinocyclicin gene clusters encoded by different *L. raffinolactis* strains.

A phylogenetic tree of the *L. raffinolactis* strains was created from the core gene alignment output obtained with Roary. The results reveal that the strain *L. raffinolactis* APC 3967 is more related to strains Lr_19_5, ERR5094873, 3042, Lr_19_14, Lr_19_4S, WiKim0068, and Lr_19_7, compared to other genomes examined (Fig. 8A). From these results, all *L. raffinolactis* genomes were investigated for the presence of the raffinocyclicin gene cluster to determine if there is a correlation between the strain location on the phylogenetic tree and bacteriocin production. Four of the 15 genomes (Lr_18_12S, 3039, ATCC 43920, and Lr_19_14S) encode the circular bacteriocin gene cluster. However, no correlation was found between the presence of the raffinocyclicin gene cluster and strain location in the phylogenetic tree (Fig. 8A).

The four strains encode an identical raffinocyclicin core peptide, but differences in the gene cluster can be observed in some strains (Fig. 8B). The strain *L. raffinolactis* ATCC 43920 seems to possess a shorter gene cluster with a truncated version of the gene *rafT2* and an absence of the genes *rafI* and *rafX*. The strains *L. raffinolactis* 3039 and Lr_18_12S have an additional open reading frame (ORF) in the gene cluster with no NCBI database hits. No other modifications were observed in the other strains.

## DISCUSSION

The circular bacteriocins are a group of peptides that have been gaining increased attention in the last few years, with the discovery of new representatives and increased interest in their potential biotechnological applications. This class of peptides has an unusual post-translational modification, namely head-to-tail cyclization, which leads to higher stability of the peptide (against pH, temperature, and proteases, among other conditions) compared to linear peptides (28). These properties make them desirable candidates for drug design and as biopreservatives. However, this group of peptides is still underexplored compared to other bacteriocin classes, with only 23 such peptides reported in the literature to date. This work presents a novel circular bacteriocin named raffinocyclicin, the first reported bacteriocin for the *L. raffinolactis* species.

Regarding the antimicrobial activity, the strain *L. raffinolactis* APC 3967 has a broad spectrum of activity, inhibiting most of the indicators used for the spot-on-lawn assay (22/31). The highest inhibitory activity was found against *L. lactis* and *C. perfringens*. It is important to highlight that *L. lactis* is a good indicator in terms of bioprospecting for circular bacteriocin producers due to its sensitivity to diverse circular bacteriocins, such as carnocyclin A (29); pumilarin (30), plantaricyclin A (31), uberolysin (32), garvicin ML (27), plantacyclin B12AG (20), leucocyclicin Q, and lactocyclicin Q (33). Indeed, the narrow-spectrum plantaricyclin A inhibited all five strains of *L. lactis* tested, emphasizing the utility of this species as an indicator in future screens for circular bacteriocins (31). In this study, *L. raffinolactis* APC 3967 could also inhibit different pathogen species, such as *C. perfringens*, *L. monocytogenes*, *E. faecium*, *E. faecalis*, and *S. agalactiae*. The inhibitory spectrum of this bacteriocin suggests its potential for application as a food biopreservative or as an antimicrobial agent in the clinic.

The proteinaceous nature of the antimicrobial activity of *L. raffinolactis* APC 3967 was confirmed following the loss of over 90% of its activity when treated with proteinase K. Furthermore, the inhibitory activity remained unchanged after NaOH treatment, ruling out that the activity was a consequence of organic acids. Exposure to trypsin revealed that the antimicrobial activity has low sensitivity to this enzyme, given that 73% of residual activity remained following treatment. Interestingly, leucocyclicin Q, lactocyclicin Q (33), and garvicin ML (27) were resistant to proteolytic activity; pumilarin presented a low-sensitivity profile (30); and carnocyclicin A had its antimicrobial activity completely abolished by protease treatment (29). This proteolytic resistance of some circular bacteriocins against proteases is not due to the lack of cleavage sites but probably due to the globular three-dimensional conformation that makes such sites inaccessible for the enzyme (27, 31).

Analysis of the raffinocyclicin gene cluster revealed 10 ORFs, 5 genes downstream of the core peptide and 4 genes upstream of it. All predicted genes necessary for bacteriocin biosynthesis are present in the cluster, such as the precursor peptide (*rafA*), a dedicated immunity protein (*rafI*), a membrane protein containing a DUF95 domain (*rafB*), an ABC transporter (*rafX*), and membrane proteins (*raf T1* and *rafT2*) (18). However, a putative three-component ABC transporter (*rafCDE*) is also encoded and is most probably associated with an accessory function as a secondary transporter. This hypothesis is supported by studies with garvicin ML, where the deletion of the corresponding genes in the operon (*garFGH*) did not affect bacteriocin production or immunity (34). For the corresponding genes of enterocin AS-48 (as-48*EFGH*) and carnocyclin A (*cclEFGH*), deletion leads to a reduction of bacteriocin immunity and/or production (35, 36). The most unusual gene found in the raffinocyclicin gene cluster is the gene *rafR*. Another example in this class of peptide of a putative transcriptional regulator was the protein LcyR encoded by a gene present in the gene cluster of leucocyclicin Q (37). The putative product of this gene is a positive transcriptional regulator of the MutR family, which is found in some bacteriocins produced by *Streptococcus mutans* to ensure the transcription of the core peptide and other genes of the cluster (38). These data suggest that the gene *rafR* could play a significant role in raffinocyclicin biosynthesis.

The alignment of the mature sequence of all circular bacteriocins (Fig. 6A) revealed a pattern in that the majority of the positively charged amino acids (lysine, arginine, and histidine) are grouped in one region of the peptide. This disproportional positive charge distribution through the peptide was also observed for enterocin AS-48. Previous studies proposed that the positive charge cluster plays a major role in the binding of the peptide to the membrane and its inhibitory activity (28). Similar asymmetrical distribution can be found in all circular bacteriocins, which leads us to propose that this region probably first interacts with the cellular surface of the target microorganism. This information could help better understand the mechanism of action of circular bacteriocins, and it should be further investigated.

A two-step method was applied to purify the peptide. Due to the lack of activity in the cell-free supernatant, the cell pellet was used as the primary source for purification. The masses found in the colony mass spectrum and purified peptide spectrum are both 6,092 ±1 Da, which confirms the hypothesis that raffinocyclicin has a three amino acid leader sequence (MFD) and that it undergoes head-to-tail circularization leading to a loss of 18 Da (6,110 − 18 = 6,092 Da). Future analysis must be done in order to support the hypothesis of the circularization event proposed from the purified bacteriocin mass spectrum.

The lack of activity in the CFSN and the activity associated with the cell extract (Fig. 1) suggest that the majority of the bacteriocin is associated with the cell. A similar phenotype has been observed previously for the lantibiotic bovicin HC5 (39). This characteristic could lead to a more stable antimicrobial activity of the bacteriocin in a complex ecosystem, such as the gut, with cell-associated peptides displaying greater resistance to proteases and peptidases produced by competing microorganisms (39). However, it is important to highlight that the circular bacteriocins present high resistance to those enzymes, as already stated. However, this hypothesis needs further evaluation for this group of peptides.

Another example of this behavior was observed with listeriolysin S, where the bacteriocin remains associated with the cell and only displayed its inhibitory activity in a contact-dependent manner. Unlike raffinocyclicin and bovicin HC5, listeriolysin S does not show inhibitory activity when not associated with the membrane of metabolically active producing bacterial cells (40). Altogether, those results propose the concept that some prokaryotes could use bacteriocins for close-contact combat in a competitive environment. This hypothesis needs to be further investigated for circular bacteriocins.

No correlation was found between the alignment of core genes and bacteriocin presence on the *L. raffinolactis* genomes, which leads to the conclusion that the gene cluster could be encoded by a mobile genetic element, such as a plasmid, which is the case of the strain APC 3967. The results show two positive hits with plasmids of the strains Lr_19_4S (query cover 36%; identity 99.96%; CP050535.1) and Lr_18_12S (query cover 22%; identity 97.80%; CP047632.1). Both strains, Lr_19_4S and Lr_18_12S, also present the complete raffinocyclicin gene cluster (Fig. 8B). The presence of the gene clusters of circular bacteriocins on plasmids has already been reported in the literature, such as enterocin AS-48, acidocin B, aureocyclicin 4185, paracyclicin, gassericin A, plantaricyclin A, and plantacyclin B12AG (41).

### Conclusion

Raffinocyclicin is a novel circular bacteriocin produced by the strain *L. raffinolactis* APC 3967 with a broad inhibitory spectrum, which undergoes head-to-tail cyclization between Leu1 and Trp61 and has a molecular mass of 6,092 Da. The plasmid-encoded gene cluster of this peptide includes 10 ORFs, and one of them, an unusual gene, *rafR*, encodes a putative-positive transcriptional regulator. The production of raffinocyclicin by a LAB opens possibilities for biotechnological applications in the food industry due to its GRAS status, either as a starter or safety culture in fermentation processes or as a purified biopreservative. More studies are necessary to evaluate this feature entirely. This is the first report of a bacteriocin produced by the *L. raffinolactis* species.

## MATERIALS AND METHODS

### Antimicrobial activity

The antimicrobial activity of the strain *L. raffinolactis* APC 3967 was evaluated on M17 medium by the agar-spot test as described by Giambiagi-deMarval et al. (42) with minor modifications (42). Briefly, 5 µL of an overnight growth suspension of the producer strain was spotted on the surface of the agar plate and incubated overnight at 30°C. Cells were inactivated by chloroform and overlaid with 3 mL soft agar (0.75% wt/vol) containing the target strain (∼7.0 log CFU/mL), and these plates were incubated overnight. The growth conditions of every target strain used in this assay are outlined in Table 1. The interpretation of inhibition was performed by measuring the true halo of the inhibitory zone (total halo – spot size = true halo) and was classified into one of four groups: (i) no inhibition (0 mm); (ii) weak inhibition (0.5–5 mm); (iii) moderate inhibition (>5–≤10 mm); and (iv) strong inhibition (>10 mm). These assays were performed in triplicate.

### Evaluation of the effects of proteases and NaOH on the antimicrobial activity

The effects of proteinase K (1 mg/mL; Sigma-Aldrich, St Louis, USA), trypsin (1 mg/mL; Merck KgaA, Darmstad, DE), and 0.2 M NaOH on antimicrobial activity were evaluated on agar plates by the methods described previously (42). Briefly, 5 µL of an overnight growth suspension of the strain *L. raffinolactis* APC 3967 was spotted four times on a plate and incubated at optimal conditions. Afterward, the cells were inactivated by chloroform, and 40 µL of the corresponding treatment (protease or NaOH 0.2 M) was spotted around the producer strain. The control was treated with 10 mM PBS, pH 7.4. The plate was incubated at 37°C for 4 h to favor protease activity. The plate was then overlaid with *L. lactis* HP and incubated at 30°C for 24 h. Results were interpreted by measuring the true halo of the sample, and the following equation calculated the final values of the residual activity: (true halo of treated condition/true halo of the control) × 100 = residual activity (%).

### Colony MALDI-TOF mass spectrometry

To determine the molecular mass of the bacteriocin produced by the strain, colonies isolated from the plate were mixed with 50 µL 70% propan-2-ol (IPA) 0.1% TFA, vortexed three times, and centrifuged at 15,000 rpm (21,000 rcf) for 20 seconds. MALDI-TOF mass spectrometry was performed on the cell-free extract using an Ultraflex MALDI-TOF mass spectrometer (Bruker, Bremen, Germany). Specifically, 0.5 µL aliquot of matrix solution [α-cyano 4-hydroxy cinnamic acid, 10 mg/mL in acetonitrile-0.1% (vol/vol) trifluoroacetic acid] was deposited onto the target and left for 20 seconds before being removed. The residual solution was air dried, and 0.5 µL sample solution was deposited onto the pre-coated sample spot. Furthermore, 0.5 µL of matrix solution was added to the deposited sample and allowed to air-dry. The sample was subsequently analyzed in positive-ion reflectron mode, and peptide masses were compared to a bacteriocin database to identify putative bacteriocins (43).

### Genome sequencing and bioinformatics analysis

DNA extraction and whole-genome sequencing of strain were performed by MicrobesNG (https://microbesng.com/). The Illumina and Oxford Nanopore platforms were applied to obtain the short- and long-sequence reads, respectively. *De novo* genome assembly of the circular genome of the strain was performed with Unicycler (v0.5.0) using short reads and long reads (44). The assembled genome was annotated using NCBI PGAP (45). The coverage of the short- and long-read sequence was done with Bowtie2 (v2.3.4.1) and minimap2 (v2.17), respectively, with Samtools (v1.7) (46–48). The presence of antimicrobial peptide gene clusters was determined using BAGEL4 (http://bagel.molgenrug.nl) (49) and antiSMASH v.7.0 (https://antismash.secondarymetabolites.org) (50). The identified open reading frame sequences were further investigated against the NCBI database using the BLAST online platform (https://www.ncbi.nlm.nih.gov/) to predict the putative functions of genes associated with bacteriocin biosynthesis (51). Molecular mass, isoelectric points aliphatic index (AI), and grand average of hydropathicity were determined using ProtParam (https://web.expasy.org/protparam/) (52). The program PSORTb v3.0.3 was applied to predict the putative cellular localization of the proteins (53).

### Circular bacteriocin core peptide alignment

The core peptide sequences of all 23 circular bacteriocins and raffinocyclicin were aligned using MAFFT v7.4.89 (54). The permutation of the three amino acids, from the beginning of the sequence to the end of the sequence, for the peptides that belong to group II (acidocin B, butyrivibriocin AR10, gassericin A, paracyclicin, plantacyclin B21AG, and plantaricyclin A) was done to improve the alignment between the sequences (20). MEGA X was used to construct the phylogram using the maximum likelihood method using the Jones-Thornton-Taylor model (55, 56). The resulting phylogram was visualized using the Interactive Tree of Life (iTOL) v6.7.4 (https://itol.embl.de/) (57).

### Bacteriocin purification

A 900 mL culture of *L. raffinolactis* APC 3967 was grown overnight at 30°C in M17 broth. The culture was centrifuged at 8,280 g for 20 minutes at 10°C, and cells were separated from the supernatant. The cell pellet was resuspended in 200 mL of 75% propan-2-ol and stirred at room temperature for 3–4 hours to extract the bacteriocin from the cell mass. The suspension was centrifuged at 8,280 g for 20 minutes, and the supernatant was retained. The IPA from the supernatant was evaporated using a rotary evaporator (Buchi Labortechnik AG, Flawil, Switzerland), and the sample was applied to a 5-g (20 mL) Strata C18-E SPE column (Phenomenex, Cheshire, UK) pre-equilibrated with methanol and water. The column was washed with 40 mL of 40% ethanol, and the inhibitory activity was eluted in 30 mL IPA. The organic solvent was removed from the sample via rotatory evaporation. The concentrated sample was applied to a semi-preparative Jupiter C4 (10 × 250 mm, 300 Å, 5 µm) RP-HPLC column (Phenomenex, Cheshire, UK) running an acetonitrile and propan-2-ol gradient described as follows: 5%–55% buffer B and 0%–5% buffer C over 25 minutes followed by 55%–19% buffer B and 5%–65% buffer C over 60 minutes, 19%–5% buffer B and 65%–95% buffer C over 5 minutes where buffer B is 90% acetonitrile 0.1% TFA and buffer C is 90% propan-2-ol 0.1% TFA. Eluent was monitored at 214 nm, and fractions were collected at 1-minute intervals. Fractions were assayed on *L. lactis* HP indicator plates, and active fractions were assayed for the antimicrobial mass of interest using MALDI TOF mass spectrometry.

### Pangenome analysis

The pangenome analysis was performed using 16 genomes from *L. raffinolactis* (including *L. raffinolactis* APC 3967) and 1 genome of a closely related species, *Lactococcus laudensis* DSM 28961, as an outlier. For this purpose, all genomes were annotated with Prokka v1.14.6 (58) and processed with Roary v3.13.0 (option “-i 95 cd 100”) (59). The core gene alignment was performed with MAFFT v7.4.89 (54) and FASTTREE v2.1.10 (60) to construct an approximate maximum likelihood phylogenetic tree. The resulting phylogram was visualized with iTOL v6.7.4 online platform (57).

### Statistical analysis

When relevant, the data were expressed as the mean value ± standard deviation. The data were analyzed by Microsoft Excel.

## Data Availability

The complete genome data (chromosome and three plasmids) of the strain *L. raffinolactis* APC 3967 are available in the GenBank/EMBL under accession no. CP147857-CP147860.

## References

[1] Guzik P, Szymkowiak A, Kulawik P, Zając M. 2022. Consumer attitudes towards food preservation methods. Foods 11:1349. doi:10.3390/foods11091349 35564072 PMC9099755

[2] Bhattacharya D, Nanda PK, Pateiro M, Lorenzo JM, Dhar P, Das AK. 2022. Lactic acid bacteria and bacteriocins: novel biotechnological approach for biopreservation of meat and meat products. Microorganisms 10:2058. doi:10.3390/microorganisms10102058 36296334 PMC9611938

[3] Mathur H, Beresford TP, Cotter PD. 2020. Health benefits of lactic acid bacteria (LAB) fermentates. Nutrients 12:1679. doi:10.3390/nu12061679 32512787 PMC7352953

[4] Chikindas ML, Weeks R, Drider D, Chistyakov VA, Dicks LM. 2018. Functions and emerging applications of bacteriocins. Curr Opin Biotechnol 49:23–28. doi:10.1016/j.copbio.2017.07.011 28787641 PMC5799035

[5] de Freire Bastos M do C, Miceli de Farias F, Carlin Fagundes P, Varella Coelho ML. 2020. Staphylococcins: an update on antimicrobial peptides produced by staphylococci and their diverse potential applications. Appl Microbiol Biotechnol 104:10339–10368. doi:10.1007/s00253-020-10946-9 33128614

[6] Rea MC, Ross RP, Cotter PD, Hill C. 2011. Classification of bacteriocins from Gram-positive bacteria, p 29–74. In Drider D, Rebuffat R (ed), Prokaryotic antimicrobial peptides. Springer, New York.

[7] Acedo JZ, Chiorean S, Vederas JC, van Belkum MJ. 2018. The expanding structural variety among bacteriocins from Gram-positive bacteria. FEMS Microbiol Rev 42:805–828. doi:10.1093/femsre/fuy033 30085042

[8] Newstead LL, Varjonen K, Nuttall T, Paterson GK. 2020. Staphylococcal-produced bacteriocins and antimicrobial peptides: their potential as alternative treatments for Staphylococcus aureus infections. Antibiotics (Basel) 9:40. doi:10.3390/antibiotics9020040 31973108 PMC7168290

[9] Sugrue I, Ross RP, Hill C. 2024. Bacteriocin diversity, function, discovery and application as antimicrobials. Nat Rev Microbiol. doi:10.1038/s41579-024-01045-x PMC7616364 38730101

[10] Cotter PD, Hill C, Ross RP. 2005. Bacteriocins: developing innate immunity for food. Nat Rev Microbiol 3:777–788. doi:10.1038/nrmicro1273 16205711

[11] Lahiri D, Nag M, Dutta B, Sarkar T, Pati S, Basu D, Abdul Kari Z, Wei LS, Smaoui S, Wen Goh K, Ray RR. 2022. Bacteriocin: a natural approach for food safety and food security. Front Bioeng Biotechnol 10:1005918. doi:10.3389/fbioe.2022.1005918 36353741 PMC9637989

[12] Gálvez A, Abriouel H, López RL, Ben Omar N. 2007. Bacteriocin-based strategies for food biopreservation. Int J Food Microbiol 120:51–70. doi:10.1016/j.ijfoodmicro.2007.06.001 17614151

[13] O’Connor PM, Kuniyoshi TM, Oliveira RP, Hill C, Ross RP, Cotter PD. 2020. Antimicrobials for food and feed; a bacteriocin perspective. Curr Opin Biotechnol 61:160–167. doi:10.1016/j.copbio.2019.12.023 31968296

[14] Carlin Fagundes P, Miceli de Farias F, Cabral da Silva Santos O, Souza da Paz JA, Ceotto-Vigoder H, Sales Alviano D, Villela Romanos MT, de Freire Bastos M do C. 2016. The four-component aureocin A70 as a promising agent for food biopreservation. Int J Food Microbiol 237:39–46. doi:10.1016/j.ijfoodmicro.2016.08.017 27543814

[15] Abriouel H, Lucas R, Ben Omar N, Valdivia E, Maqueda M, Martínez-Cañamero M, Gálvez A. 2005. Enterocin AS-48RJ: a variant of enterocin AS-48 chromosomally encoded by Enterococcus faecium RJ16 isolated from food. Syst Appl Microbiol 28:383–397. doi:10.1016/j.syapm.2005.01.007 16094865

[16] Potter A, Ceotto H, Coelho MLV, Guimarães AJ, Bastos M do C de F. 2014. The gene cluster of aureocyclicin 4185: the first cyclic bacteriocin of Staphylococcus aureus. Microbiology 160:917–928. doi:10.1099/mic.0.075689-0 24574434

[17] Collins FWJ, O’Connor PM, O’Sullivan O, Gómez-Sala B, Rea MC, Hill C, Ross RP. 2017. Bacteriocin gene-trait matching across the complete Lactobacillus pan-genome. Sci Rep 7:3481. doi:10.1038/s41598-017-03339-y 28615683 PMC5471241

[18] Perez RH, Zendo T, Sonomoto K. 2018. Circular and leaderless bacteriocins: biosynthesis, mode of action, applications, and prospects. Front Microbiol 9:2085. doi:10.3389/fmicb.2018.02085 30233551 PMC6131525

[19] Kurata A, Yamaguchi T, Kira M, Kishimoto N. 2019. Characterization and heterologous expression of an antimicrobial peptide from Bacillus amyloliquefaciens CMW1. Biotechn Biotechnological Eq 33:886–893. doi:10.1080/13102818.2019.1627246

[20] Golneshin A, Gor M-C, Williamson N, Vezina B, Van TTH, May BK, Smith AT. 2020. Discovery and characterisation of circular bacteriocin plantacyclin B21AG from Lactiplantibacillus plantarum B21. Heliyon 6:e04715. doi:10.1016/j.heliyon.2020.e04715 32904251 PMC7452424

[21] Xin B, Liu H, Zheng J, Xie C, Gao Y, Dai D, Peng D, Ruan L, Chen H, Sun M. 2020. In silico analysis highlights the diversity and novelty of circular bacteriocins in sequenced microbial genomes. mSystems 5:e00047-20. doi:10.1128/mSystems.00047-20 32487738 PMC8534725

[22] Xin B, Xu H, Liu H, Liu S, Wang J, Xue J, Zhang F, Deng S, Zeng H, Zeng X, Xu D, Zhao Y, Li F, Wang G. 2021. Identification and characterization of a novel circular bacteriocin, bacicyclicin XIN-1, from Bacillus sp. XIN1. Food Control 121:107696. doi:10.1016/j.foodcont.2020.107696

[23] Kita K, Yoshida S, Masuo S, Nakamura A, Ishikawa S, Yoshida K-I. 2023. Genes encoding a novel thermostable bacteriocin in the thermophilic bacterium Aeribacillus pallidus PI8. J Appl Microbiol 134:lxad293. doi:10.1093/jambio/lxad293 38040658

[24] Lafuente I, Sevillano E, Peña N, Cuartero A, Hernández PE, Cintas LM, Muñoz-Atienza E, Borrero J. 2024. Production of pumilarin and a novel circular bacteriocin, altitudin A, by Bacillus altitudinis ECC22, a soil-derived bacteriocin producer. Int J Mol Sci 25:2020. doi:10.3390/ijms25042020 38396696 PMC10888436

[25] Peña N, Bland MJ, Sevillano E, Muñoz-Atienza E, Lafuente I, Bakkoury ME, Cintas LM, Hernández PE, Gabant P, Borrero J. 2022. In vitro and in vivo production and split-intein mediated ligation (SIML) of circular bacteriocins. Front Microbiol 13:1052686. doi:10.3389/fmicb.2022.1052686 36452926 PMC9703936

[26] Martínez B, Fernández M, Suárez JE, Rodríguez A. 1999. Synthesis of lactococcin 972, a bacteriocin produced by Lactococcus lactis IPLA 972, depends on the expression of a plasmid-encoded bicistronic operon the GenBank accession number for the sequence reported in this paper is AJ002203. Microbiology 145:3155–3161. doi:10.1099/00221287-145-11-3155 10589723

[27] Borrero J, Brede DA, Skaugen M, Diep DB, Herranz C, Nes IF, Cintas LM, Hernández PE. 2011. Characterization of garvicin ML, a novel circular bacteriocin produced by Lactococcus garvieae DCC43, isolated from mallard ducks (Anas platyrhynchos). Appl Environ Microbiol 77:369–373. doi:10.1128/AEM.01173-10 21057028 PMC3019728

[28] Cebrián R, Martínez-García M, Fernández M, García F, Martínez-Bueno M, Valdivia E, Kuipers OP, Montalbán-López M, Maqueda M. 2023. Advances in the preclinical characterization of the antimicrobial peptide AS-48. Front Microbiol 14:1110360. doi:10.3389/fmicb.2023.1110360 36819031 PMC9936517

[29] Martin-Visscher LA, van Belkum MJ, Garneau-Tsodikova S, Whittal RM, Zheng J, McMullen LM, Vederas JC. 2008. Isolation and characterization of carnocyclin A, a novel circular bacteriocin produced by Carnobacterium maltaromaticum UAL307. Appl Environ Microbiol 74:4756–4763. doi:10.1128/AEM.00817-08 18552180 PMC2519327

[30] van Heel AJ, Montalban-Lopez M, Oliveau Q, Kuipers OP. 2017. Genome-guided identification of novel head-to-tail cyclized antimicrobial peptides, exemplified by the discovery of pumilarin. Microb Genom 3:e000134. doi:10.1099/mgen.0.000134 29177092 PMC5695211

[31] Borrero J, Kelly E, O’Connor PM, Kelleher P, Scully C, Cotter PD, Mahony J, van Sinderen D. 2018. Plantaricyclin a, a novel circular bacteriocin produced by Lactobacillus plantarum NI326: purification, characterization, and heterologous production. Appl Environ Microbiol 84:e01801-17. doi:10.1128/AEM.01801-17 29030449 PMC5734033

[32] Wirawan RE, Swanson KM, Kleffmann T, Jack RW, Tagg JR. 2007. Uberolysin: a novel cyclic bacteriocin produced by Streptococcus uberis. Microbiology 153:1619–1630. doi:10.1099/mic.0.2006/005967-0 17464077

[33] Masuda Y, Ono H, Kitagawa H, Ito H, Mu F, Sawa N, Zendo T, Sonomoto K. 2011. Identification and characterization of leucocyclicin Q, a novel cyclic bacteriocin produced by Leuconostoc mesenteroides TK41401. Appl Environ Microbiol 77:8164–8170. doi:10.1128/AEM.06348-11 21948835 PMC3209004

[34] Gabrielsen C, Brede DA, Salehian Z, Nes IF, Diep DB. 2014. Functional genetic analysis of the GarML gene cluster in Lactococcus garvieae DCC43 gives new insights into circular bacteriocin biosynthesis. J Bacteriol 196:911–919. doi:10.1128/JB.01115-13 24336941 PMC3957696

[35] Diaz M, Valdivia E, Martínez-Bueno M, Fernández M, Soler-González AS, Ramírez-Rodrigo H, Maqueda M. 2003. Characterization of a new operon, as-48EFGH, from the as-48 gene cluster involved in immunity to enterocin AS-48. Appl Environ Microbiol 69:1229–1236. doi:10.1128/AEM.69.2.1229-1236.2003 12571051 PMC143590

[36] van Belkum MJ, Martin-Visscher LA, Vederas JC. 2010. Cloning and characterization of the gene cluster involved in the production of the circular bacteriocin carnocyclin A. Probiotics Antimicro Prot 2:218–225. doi:10.1007/s12602-010-9056-1 26781316

[37] Mu F, Masuda Y, Zendo T, Ono H, Kitagawa H, Ito H, Nakayama J, Sonomoto K. 2014. Biological function of a DUF95 superfamily protein involved in the biosynthesis of a circular bacteriocin, leucocyclicin Q. J Biosci Bioeng 117:158–164. doi:10.1016/j.jbiosc.2013.06.023 23906710

[38] Merritt J, Qi F. 2012. The mutacins of Streptococcus mutans: regulation and ecology. Mol Oral Microbiol 27:57–69. doi:10.1111/j.2041-1014.2011.00634.x 22394465 PMC3296966

[39] Xavier BM, Houlihan AJ, Russell JB. 2008. The activity and stability of cell-associated activity of bovicin HC5, a bacteriocin from Streptococcus bovis HC5. FEMS Microbiol Lett 283:162–166. doi:10.1111/j.1574-6968.2008.01155.x 18445020

[40] Meza-Torres J, Lelek M, Quereda JJ, Sachse M, Manina G, Ershov D, Tinevez J-Y, Radoshevich L, Maudet C, Chaze T, Giai Gianetto Q, Matondo M, Lecuit M, Martin-Verstraete I, Zimmer C, Bierne H, Dussurget O, Cossart P, Pizarro-Cerdá J. 2021. Listeriolysin S: a bacteriocin from Listeria monocytogenes that induces membrane permeabilization in a contact-dependent manner. Proc Natl Acad Sci U S A 118:e2108155118. doi:10.1073/pnas.2108155118 34599102 PMC8501752

[41] Vezina B, Rehm BHA, Smith AT. 2020. Bioinformatic prospecting and phylogenetic analysis reveals 94 undescribed circular bacteriocins and key motifs. BMC Microbiol 20:77. doi:10.1186/s12866-020-01772-0 32252629 PMC7132975

[42] Giambiagi-Marval M, Mafra MA, Penido EGC, Bastos MCF. 1990. Distinct groups of plasmids correlated with bacteriocin production in Staphylococcus aureus. J Gen Microbiol 136:1591–1599. doi:10.1099/00221287-136-8-1591 2175767

[43] Collins FWJ, O’Connor PM, O’Sullivan O, Rea MC, Hill C, Ross RP. 2016. Formicin – a novel broad-spectrum two-component lantibiotic produced by Bacillus paralicheniformis APC 1576. Microbiology 162:1662–1671. doi:10.1099/mic.0.000340 27450592

[44] Wick RR, Judd LM, Gorrie CL, Holt KE. 2017. Unicycler: resolving bacterial genome assemblies from short and long sequencing reads. PLoS Comput Biol 13:e1005595. doi:10.1371/journal.pcbi.1005595 28594827 PMC5481147

[45] Li W, O’Neill KR, Haft DH, DiCuccio M, Chetvernin V, Badretdin A, Coulouris G, Chitsaz F, Derbyshire MK, Durkin AS, Gonzales NR, Gwadz M, Lanczycki CJ, Song JS, Thanki N, Wang J, Yamashita RA, Yang M, Zheng C, Marchler-Bauer A, Thibaud-Nissen F. 2021. RefSeq: expanding the prokaryotic genome annotation pipeline reach with protein family model curation. Nucleic Acids Research 49:D1020–D1028. doi:10.1093/nar/gkaa1105 33270901 PMC7779008

[46] Li H. 2018. Minimap2: pairwise alignment for nucleotide sequences. Bioinformatics 34:3094–3100. doi:10.1093/bioinformatics/bty191 29750242 PMC6137996

[47] Langmead B, Salzberg SL. 2012. Fast gapped-read alignment with Bowtie 2. Nat Methods 9:357–359. doi:10.1038/nmeth.1923 22388286 PMC3322381

[48] Li H, Handsaker B, Wysoker A, Fennell T, Ruan J, Homer N, Marth G, Abecasis G, Durbin R, 1000 Genome Project Data Processing Subgroup. 2009. The sequence alignment/map format and SAMtools. Bioinformatics 25:2078–2079. doi:10.1093/bioinformatics/btp352 19505943 PMC2723002

[49] van Heel AJ, de Jong A, Song C, Viel JH, Kok J, Kuipers OP. 2018. BAGEL4: a user-friendly web server to thoroughly mine RiPPs and bacteriocins. Nucleic Acids Res 46:W278–W281. doi:10.1093/nar/gky383 29788290 PMC6030817

[50] Blin K, Shaw S, Augustijn HE, Reitz ZL, Biermann F, Alanjary M, Fetter A, Terlouw BR, Metcalf WW, Helfrich EJN, van Wezel GP, Medema MH, Weber T. 2023. antiSMASH 7.0: new and improved predictions for detection, regulation, chemical structuresand visualisation. Nucleic Acids Res 51:W46–W50. doi:10.1093/nar/gkad344 37140036 PMC10320115

[51] Altschul SF, Madden TL, Schäffer AA, Zhang J, Zhang Z, Miller W, Lipman DJ. 1997. Gapped BLAST and PSI-BLAST: a new generation of protein database search programs. Nucleic Acids Res 25:3389–3402. doi:10.1093/nar/25.17.3389 9254694 PMC146917

[52] Duvaud S, Gabella C, Lisacek F, Stockinger H, Ioannidis V, Durinx C. 2021. Expasy, the swiss bioinformatics resource portal, as designed by its users. Nucleic Acids Res 49:W216–W227. doi:10.1093/nar/gkab225 33849055 PMC8265094

[53] Yu NY, Wagner JR, Laird MR, Melli G, Rey S, Lo R, Dao P, Sahinalp SC, Ester M, Foster LJ, Brinkman FSL. 2010. PSORTb 3.0: improved protein subcellular localization prediction with refined localization subcategories and predictive capabilities for all prokaryotes. Bioinformatics 26:1608–1615. doi:10.1093/bioinformatics/btq249 20472543 PMC2887053

[54] Katoh K, Standley DM. 2013. MAFFT multiple sequence alignment software version 7: improvements in performance and usability. Mol Biol Evol 30:772–780. doi:10.1093/molbev/mst010 23329690 PMC3603318

[55] Kumar S, Stecher G, Li M, Knyaz C, Tamura K. 2018. MEGA X: molecular evolutionary genetics analysis across computing platforms. Mol Biol Evol 35:1547–1549. doi:10.1093/molbev/msy096 29722887 PMC5967553

[56] Jones DT, Taylor WR, Thornton JM. 1992. The rapid generation of mutation data matrices from protein sequences. Bioinformatics 8:275–282. doi:10.1093/bioinformatics/8.3.275 1633570

[57] Letunic I, Bork P. 2021. Interactive Tree Of Life (iTOL) v5: an online tool for phylogenetic tree display and annotation. Nucleic Acids Res 49:W293–W296. doi:10.1093/nar/gkab301 33885785 PMC8265157

[58] Seemann T. 2014. Prokka: rapid prokaryotic genome annotation. Bioinformatics 30:2068–2069. doi:10.1093/bioinformatics/btu153 24642063

[59] Page AJ, Cummins CA, Hunt M, Wong VK, Reuter S, Holden MTG, Fookes M, Falush D, Keane JA, Parkhill J. 2015. Roary: rapid large-scale prokaryote pan genome analysis. Bioinformatics 31:3691–3693. doi:10.1093/bioinformatics/btv421 26198102 PMC4817141

[60] Price MN, Dehal PS, Arkin AP. 2010. FastTree 2--approximately maximum-likelihood trees for large alignments. PLoS One 5:e9490. doi:10.1371/journal.pone.0009490 20224823 PMC2835736

